# Association between HDL levels and stroke outcomes in the Arab population

**DOI:** 10.1038/s41598-024-53613-z

**Published:** 2024-02-06

**Authors:** Aizaz Ali, Omar Obaid, Naveed Akhtar, Rahul Rao, Syed Haroon Tora, Ashfaq Shuaib

**Affiliations:** 1 Department of Neurology, University of Toledo Medical Center, 3000 Arlington Avenue, Toledo, OH 43614 USA; 2 Department of General Surgery, University of Toledo Medical Center, Toledo, OH USA; 3 Department of Neurology, Hamad Medical Corporation, Doha, Qatar; 4 Master of Public Health Candidate, Bloomberg School of Public Health, Johns Hopkins University, Baltimore, MD USA; 5 University of Alberta, Neurology, Edmonton, Canada

**Keywords:** High density lipoprotein, Stroke, Post-stroke outcomes, MACE, Cerebral infarction, Epidemiology, Diseases of the nervous system, Stroke

## Abstract

Low HDL levels are associated with an increased stroke incidence and worsened long-term outcomes. The aim of this study was to assess the relationship between HDL levels and long-term stroke outcomes in the Arab population. Patients admitted to the Qatar Stroke Database between 2014 and 2022 were included in the study and stratified into sex-specific HDL quartiles. Long-term outcomes included 90-Day modified Rankin Score (mRS), stroke recurrence, and post-stroke cardiovascular complications within 1 year of discharge. Multivariate binary logistic regression analyses were performed to identify the independent effect of HDL levels on short- and long-term outcomes. On multivariate binary logistic regression analyses, 1-year stroke recurrence was 2.24 times higher (p = 0.034) and MACE was 1.99 times higher (p = 0.009) in the low-HDL compared to the high-HDL group. Mortality at 1 year was 2.27-fold in the low-normal HDL group compared to the reference group (p = 0.049). Lower sex-specific HDL levels were independently associated with higher adjusted odds of 1-year post-stroke mortality, stroke recurrence, and MACE (*p* < 0.05). In patients who suffer a stroke, low HDL levels are associated with a higher risk of subsequent vascular complication.

## Introduction

Stroke is the second leading cause of death and a major cause of disability worldwide. Stroke is a preventable disease with multiple modifiable risk factors including but not limited to hypertension, diabetes mellitus, dyslipidemia, obesity and smoking. Several studies have attempted to understand the relationship between stroke, lipid biomarkers, and optimization of dyslipidemia management. The association between low density lipoprotein cholesterol (LDL-C) and stroke is well established, with higher levels being directly related to cerebral infarction and with lipid lowering therapy being used as a preventive strategy to mitigate stroke risk^1^. A recent meta-analysis has demonstrated a significant association between triglyceride level (TG) and ischemic stroke^2^, whereas another meta-analysis has demonstrated that a high density lipoprotein cholesterol (HDL-C) level is associated with reduced risk of total stroke and ischemic stroke (IS)^3^.

## Aims/Hypothesis

Most of the studies evaluating the effects of HDL-C on stroke have been completed in a Caucasian population. There is very little information on the long-term effects of changes in HDL on stroke outcome in the Arabic population. In this study we explored the Qatar stroke database to study the effects of various levels of serum HDL on the presentation of stroke severity, in-hospital complications and short and long-term outcomes in a well defined Qatari population.

## Methods

The Qatar stroke database prospectively collects data on stroke patients admitted to the Hamad General Hospital (HGH), the only tertiary care hospital for stroke admissions in the State of Qatar. The details of the database have previously been published^4^. This is a retrospective cohort analysis of stroke patients from the database. All patients admitted with a stroke, including ischemic stroke, stroke mimics, transient ischemic attack (TIA) and intracranial hemorrhage admitted to Hamad General Hospital (HGH), Doha, Qatar between January 1, 2014 and December 04, 2021 were available for analysis. For the purpose of this study we included only Arab patients with a confirmed diagnosis of ischemic stroke. In order to better understand the relationship between HDL and stroke, we excluded patients with missing data on HDL levels.

Dyslipidemia was defined as a low density lipoprotein cholesterol (LDL) level ≥ 3.62 mmol/L, high density lipoprotein cholesterol (HDL) level ≤ 1.03 mmol/L, triglycerides ≥ 1.69 mmol/L, or current treatment with a cholesterol-lowering drug^5^. Patients were stratified into 4 sex-specific quartiles based on their HDL levels on admission: Low (L) males: ≤ 0.80 mmol/dL, females: ≤ 1.00 mmol/dL; Low-normal (LN) males: 0.80–1.00 mmol/dL, females: 1.00–1.30 mmol/dL; High-normal (HN) males: 1.00–1.12 mmol/dL, females: 1.30–1.60 mmol/dL; and High (H) males: > 1.12 mmol/dL, females: > 1.60 mmol/dL.

Atrial fibrillation (AF) was diagnosed based on electrocardiographic findings on admission or on holter monitoring during hospitalization. Smoking was defined as current cigarette smoking. Diabetes was diagnosed according to the American Diabetes Association (ADA) and World Health Organization (WHO) recommendations and included patients with a previous diagnosis of diabetes mellitus (DM), on medication for DM or a HbA1c ≥ 6.5% and the diagnosis of pre-DM was based on a HbA1c of 5.7—6.4% as per 2015 ADA clinical practice recommendations^6^. Hypertension was defined as a previous systolic blood pressure ≥ 140 mm Hg or a diastolic blood pressure ≥ 90 mm Hg, or current treatment with antihypertensive drugs. Complications monitored and recorded included aspiration pneumonia, urinary tract infection, bedsores, deep venous thrombosis, and sepsis during hospitalization.

Inpatient outcome measures were National Institute of Health stroke scale (NIHSS) on admission, in-hospital mortality, Intensive are unit (ICU) admission, major complications, in-hospital interventions including thrombolysis and mechanical thrombectomy, and survivor-only hospital length of stay (LOS). Long-term outcome measures among survivors of index admission with adequate follow-up data available were mortality and modified Rankin Score (mRS) at 90 days of discharge, and stroke recurrence, post-stroke myocardial infarction (MI), post-stroke major cardiac adverse events (MACE), and post-stroke cardiac revascularization procedures within 1 year of discharge. To achieve this, the Cerner electronic medical systems were used to track patient admissions throughout the state of Qatar.

Descriptive results for all continuous variables were reported as mean ± standard deviation (SD) when normally distributed or as medians with interquartile ranges (IQR) when non-normally distributed. The distribution of continuous variables was assessed by applying Kolmogorov Smirnov tests prior to using statistical tools. Descriptive results for all categorical variables were reported as numbers and percentages. ANOVA test was used to compare normally distributed continuous variables between groups. Kruskall-Wallis h test was used to compare non-parametric continuous variables between groups. Chi-square or Fisher’s exact test were used to compare categorical variables between groups where appropriate.

Multivariate binary logistic regression analyses were performed to determine the independent effect of sex-specific TG-HDL indices on outcomes: In-hospital mortality, in-hospital major complications, 1-year stroke recurrence, 1-year post-stroke MACE, 1-year post-stroke cardiac revascularization, 90-day mortality, and 1-year mortality.

Multivariate analyses adjusted for potential confounders, including patient demographics (age, sex), emergency department vitals (systolic blood pressure), comorbidities (diabetes, hypertension, dyslipidemia, coronary artery disease, atrial fibrillation, prior stroke history, obesity, smoking status), stroke severity, and in-hospital interventions (thrombolysis or thrombectomy). Covariates for multivariate analyses were chosen from an initial bivariate analysis as well as from prior literature demonstrating effect on stroke outcomes. Alpha was set at 5% and a *p*-value less than 0.05 was considered statistically significant. All statistical analyses were performed using IBM Statistical Product and Service Solutions (SPSS) version 28. All methods were carried out in accordance with relevant guidelines and regulations. Informed consent was obtained from all subjects and/or their legal guardian(s). The study was approved by the Committee for Human Ethics Research, Academic Health Service at HMC (MRC-01-20-1135).

### Ethics statement

The study was approved by the Committee for Human Ethics Research, Academic Health Service at HMC (MRC-01-20-1135).

## Results

A total of 2089 ethnically Arab stroke patients were identified (658 L, 722 LN, 309 HN, and 400 H). Overall, the mean age was 62 ± 14 years. 1364 patients (66%) were male, 886 (43%) had BMI ≥ 30, 1403 (68%) had diabetes mellitus, 1,635 (80%) had hypertension, 1,126 (55%) had dyslipidemia, and 594 (29%) were smokers. The median TG levels were 1.4 [1.0–2.1], HDL levels 1.0 [0.8–1.2], and LDL levels was 2.8 [2.0–3.5]. The proportion of patients with DM was highest in the Low HDL group (71.9%) as compared to High HDL group (62.1%), with a similar trend observed for Obesity—46.7% vs 34.1% respectively. CAD and smoking rates were highest in the Low HDL. Median NIHSS was the highest in the low-HDL group at 4 [2–7]. According to the TOAST classification, small vessel disease accounted for the highest proportion of strokes (50%), followed by cardioembolic strokes (21.8%), and large vessel disease (17.9%). There was no difference between HDL groups in terms of stroke etiology according to TOAST classification (Table 1).

**Table 1:** Baseline Characteristics Stratified by Sex-Specific HDL Quartiles.

Sex-specific HDL Cut-offs	Overall (n = 2089)	Low HDL (n = 658)	Low-Normal HDL (n = 722)	High-Normal HDL (n = 309)	High HDL (n = 400)	*p-*value
		M: ≤ 0.80 F: ≤ 1.00	M: 0.80–1.00 F: 1.00–1.30	M: 1.00–1.12 F: 1.30–1.60	M: > 1.12 F: > 1.60	
Baseline characteristics						
Patient demographics						
Age, years, mean ± SD	62 ± 14	61 ± 13	63 ± 14	64 ± 14	62 ± 14	0.005*
Age > 55 years, n (%)	1,489 (72.5)	445 (68.8)	525 (73.5)	228 (75.0)	291 (74.6)	0.082
Male sex, n (%)	1,364 (66.4)	409 (63.2)	438 (61.3)	180 (59.2)	337 (86.4)	< 0.001*
BMI, mean ± SD	30 ± 6	30 ± 6	30 ± 7	30 ± 6	29 ± 6	< 0.001*
Patient comorbidities						
Diabetes mellitus, n (%)	1403 (68.3)	465 (71.9)	498 (69.7)	198 (65.1)	242 (62.1)	0.005*
Hypertension, n (%)	1635 (79.6)	505 (78.1)	570 (79.8)	247 (81.3)	313 (80.3)	0.662
Atrial fibrillation, n (%)	253 (12.3)	78 (12.1)	82 (11.5)	46 (15.1)	47 (12.1)	0.431
Prior stroke history, n (%)	392 (19.1)	128 (19.8)	131 (18.3)	55 (18.1)	78 (20.0)	0.835
CAD, n (%)	395 (19.2)	151 (23.3)	127 (17.8)	44 (14.5)	73 (18.7)	0.006*
Smoking history, n (%)	594 (28.9)	221 (34.2)	195 (27.3)	75 (24.7)	103 (26.4)	0.004*
Obesity, n (%)	886 (43.1)	302 (46.7)	325 (45.5)	126 (41.4)	133 (34.1)	< 0.001*
Dyslipidemia, n (%)	1,126 (54.8)	328 (50.7)	406 (56.9)	161 (53.0)	231 (59.2)	0.028*
Admission vitals						
SBP, mm Hg, mean ± SD	153 ± 27	149 ± 28	154 ± 27	156 ± 27	153 ± 28	0.001*
DBP, mm Hg, mean ± SD	84 ± 17	83 ± 17	84 ± 17	86 ± 17	85 ± 17	0.037*
Admission labs						
RBS, mmol/L, median [IQR]	8.5 [6.2–12.9]	9.0 [6.4–13.3]	8.5 [6.3–12.8]	8.2 [5.9–12.8]	8.1 [6.2–12.5]	0.294
HbA1C, %, median [IQR]	6.9 [5.8–9.1]	7.1 [5.8–9.2]	7.1 [5.8–9.3]	6.8 [5.7–8.9]	6.5 [5.7–9.0]	0.078
TG, mmol/L, median [IQR]	1.4 [1.0–2.1]	1.9 [1.3–2.6]	1.5 [1.1–2.0]	1.2 [1.0–1.7]	1.1 [0.8–1.6]	< 0.001*
HDL, mmol/L, median [IQR]	1.0 [0.8–1.2]	0.8 [0.7–0.8]	1.0 [0.9–1.1]	1.1 [1.1–1.4]	1.3 [1.2–1.5]	< 0.001*
LDL, mmol/L, median [IQR]	2.8 [2.0–3.5]	2.5 [1.8–3.4]	2.8 [2.2–3.6]	2.8 [2.1–3.6]	2.8 [2.1–3.7]	< 0.001*
Chol, mmol/L, median [IQR]	5.6 [3.7–5.4]	4.2 [3.4–5.2]	4.6 [3.8–5.4]	4.7 [4.0–5.5]	4.8 [4.1–5.8]	< 0.001*
Stroke characteristics						
NIHSS, median [IQR]	3 [1–6]	4 [2–7]	3 [1–6]	3 [2–7]	3 [1–6]	0.004*
TOAST classification, n (%)						
Small-vessel disease	1,028 (50.0)	314 (48.5)	359 (50.3)	148 (48.7)	207 (53.1)	0.424
Large-vessel disease	368 (17.9)	125 (19.3)	131 (18.3)	45 (14.8)	67 (17.2)	
Cardioembolic stroke	448 (21.8)	141 (21.8)	147 (20.6)	80 (26.3)	80 (20.5)	
Determined origin	134 (6.5)	38 (5.9)	54 (7.6)	17 (5.6)	25 (6.4)	
Undetermined origin	77 (3.7)	29 (4.5)	23 (3.2)	14 (4.6)	11 (2.8)	

*TG* triglyceride, *HDL* high density lipoprotein, *M* males, *F* females, *SD* standard deviation, *BMI* body mass index, *CAD* coronary artery disease, *SBP* systolic blood pressure, *DBP* diastolic blood pressure, *RBS* random blood sugar, *HbA1C* glycosylated hemoglobin, *LDL* low density lipoprotein, *Chol* cholesterol, *NIHSS* National Institutes on Health Stroke Severity Scale, *TOAST* trial of ORG 10,172 in Acute Stroke Treatment Classification.

Overall, 213 (10%) patients received thrombolysis, 71 (3%) thrombectomy, 87 (4%) were admitted to ICU, 190 (9%) suffered major complications, median survivor-only LOS was 4 [2–7] days and 34 (1.6%) died during their index hospitalizations. Among 2055 survivors of index admission who had 1-year follow-up data available, 73 (3%) had a recurrent stroke (most commonly ischemic), 190 (9.2%) had MACE, 11 (0.5%) had post-stroke MI, and 13 (0.6%) had post-stroke cardiac revascularization procedure within 1 year of discharge. Mortality within 90 days of discharge was 3.4% and within one year was 4.8%.

On univariate analyses, LOS was highest for the Low-HDL group compared to all the other groups (p = 0.045). There was no statistically significant difference in the rate of thrombolysis between the different sex-specific HDL quartiles, however, thrombectomy rates in the Low-HDL group were almost twice as that of the High-HDL group (p = 0.026). Similar rates were also observed for the 1-year stroke recurrence [6.1% vs. 2.9% (p = 0.014)] and 1-year post-stroke MACE [11.7% vs. 6.2% (p = 0.021)] between the low-HDL and high-HDL group. No statistically significant difference was observed between the sex-specific HDL quartiles for the 90-day and 1-year mortality, and for the 1-year post-stroke MI and cardiac revascularization procedures (Table 2).

**Table 2:** Univariate Analysis of Outcomes Stratified by Sex-Specific HDL Quartiles.

Sex-specific HDL cut-offs	Low HDL (n = 658)	Low-Normal HDL (n = 722)	High-Normal HDL (n = 309)	High HDL (n = 400)	*p-*value
	M: ≤ 0.80 F: ≤ 1.00	M: 0.80–1.00 F: 1.00–1.30	M: 1.00–1.12 F: 1.30–1.60	M: > 1.12 F: > 1.60	
Inpatient outcomes					
LOS, d, median [IQR]	4 [3–7]	4 [2–6]	4 [2–7]	4 [2–6]	0.045*
In-hospital mortality, n (%)	11 (1.7)	8 (1.1)	5 (1.6)	10 (2.5)	0.372
Thrombolysis, n (%)	76 (11.6)	67 (9.3)	36 (11.7)	34 (8.5)	0.267
Thrombectomy, n (%)	34 (5.2)	19 (2.6)	7 (2.3)	11 (2.8)	0.026*
ICU admission, n (%)	29 (4.4)	28 (3.9)	12 (3.9)	18 (4.5)	0.936
In-hospital complications, n (%)	67 (10.2)	63 (8.7)	24 (7.8)	36 (9.0)	0.632
Post-discharge long-term outcomes					
1-y stroke recurrence, n (%)	36 (6.1)	19 (2.9)	8 (3.0)	10 (2.9)	0.014*
mRS at 90 days, median [IQR]	2 [0–3]	1 [0–3]	1 [0–3]	1 [0–3]	0.076
90-day mortality, n (%)	26 (4.8)	26 (4.4)	11 (4.4)	6 (1.9)	0.209
1-y mortality, n (%)	35 (5.9)	38 (5.9)	17 (6.3)	9 (2.6)	0.094
1-y post-stroke MACE, n (%)	76 (11.7)	61 (8.5)	29 (9.5)	24 (6.2)	0.021*
1-y post-stroke MI, n (%)	7 (1.2)	3 (0.5)	1 (0.4)	Nil	0.116
1-y post-stroke cardiac revascularization, n (%)	4 (0.7)	4 (0.6)	3 (1.1)	2 (0.6)	0.844

*HDL* high-density lipoprotein, *LOS* length of stay, *d* days, *y* year, *IQR* interquartile range, *ICU* intensive care unit, *MRS* modified Rankin Scale, *MACE* major adverse cardiac event, *MI* myocardial infarction, *M* males, *F* females.

Long-term outcomes and in-hospital LOS were assessed among survivors of index hospital admission.

**Figure 1:**
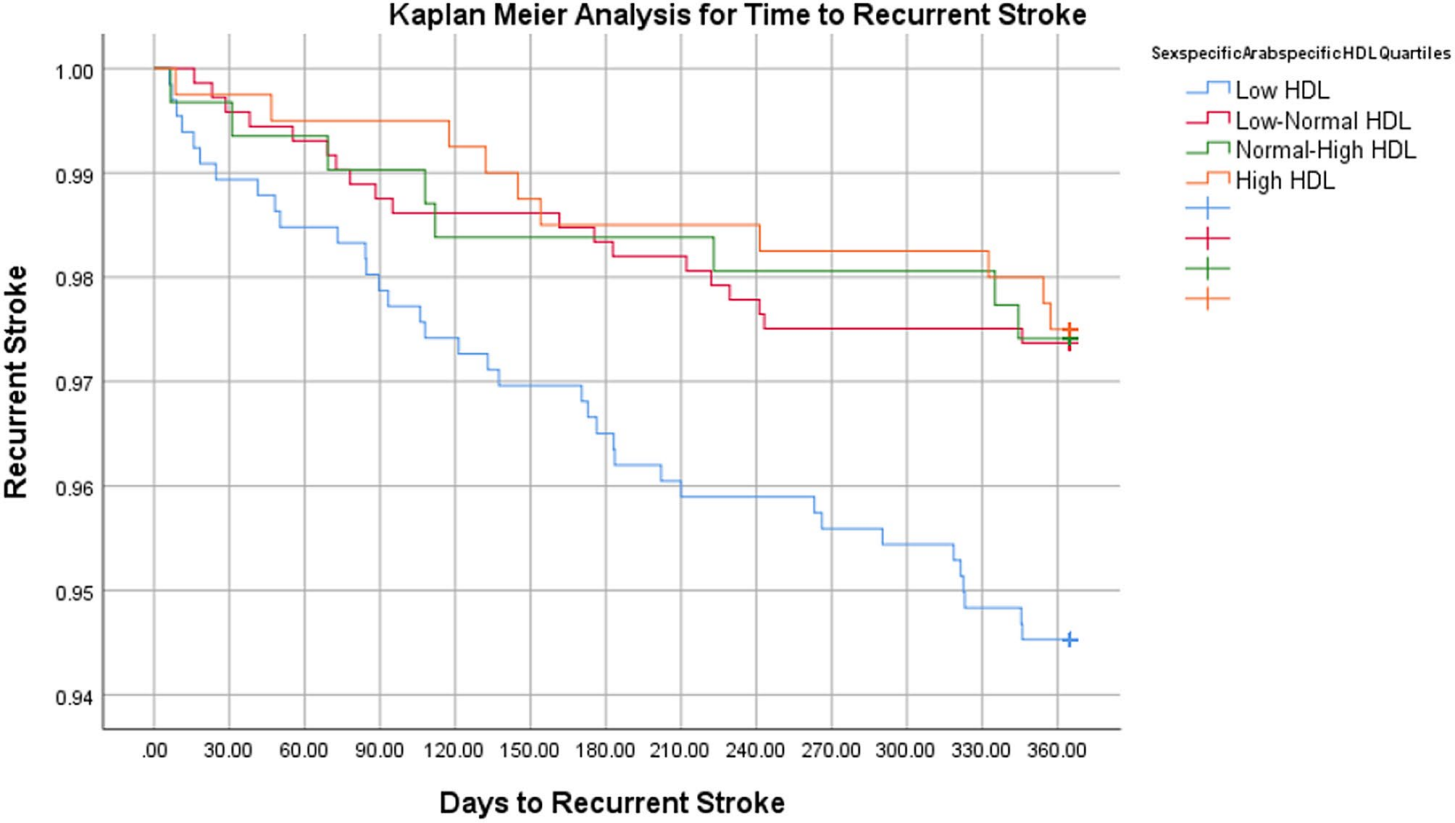
Kaplan meier curve for time to recurrent stroke.

**Figure 2:**
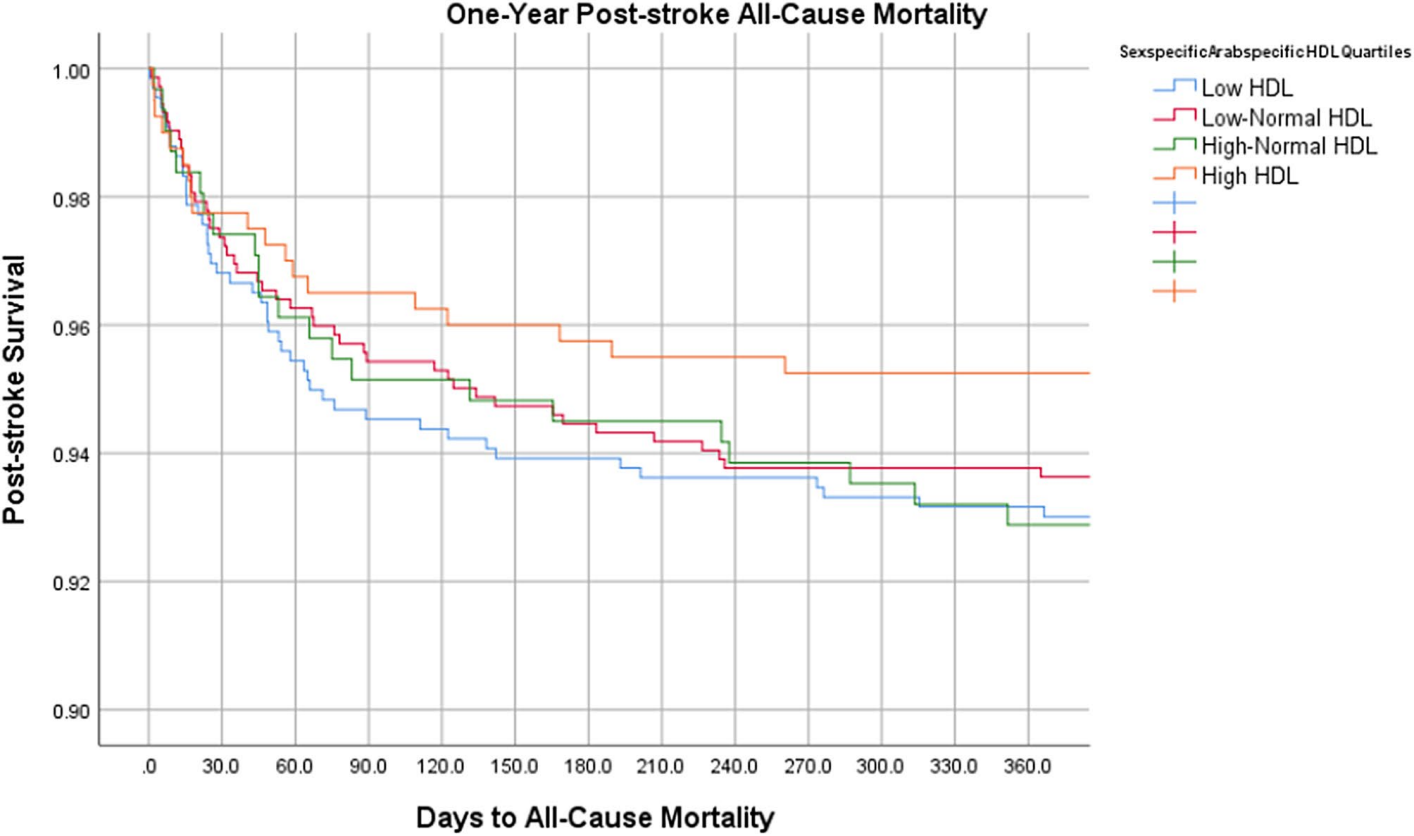
Kaplan meier curve for 1-year post-stroke all cause mortality.

**Figure 3:**
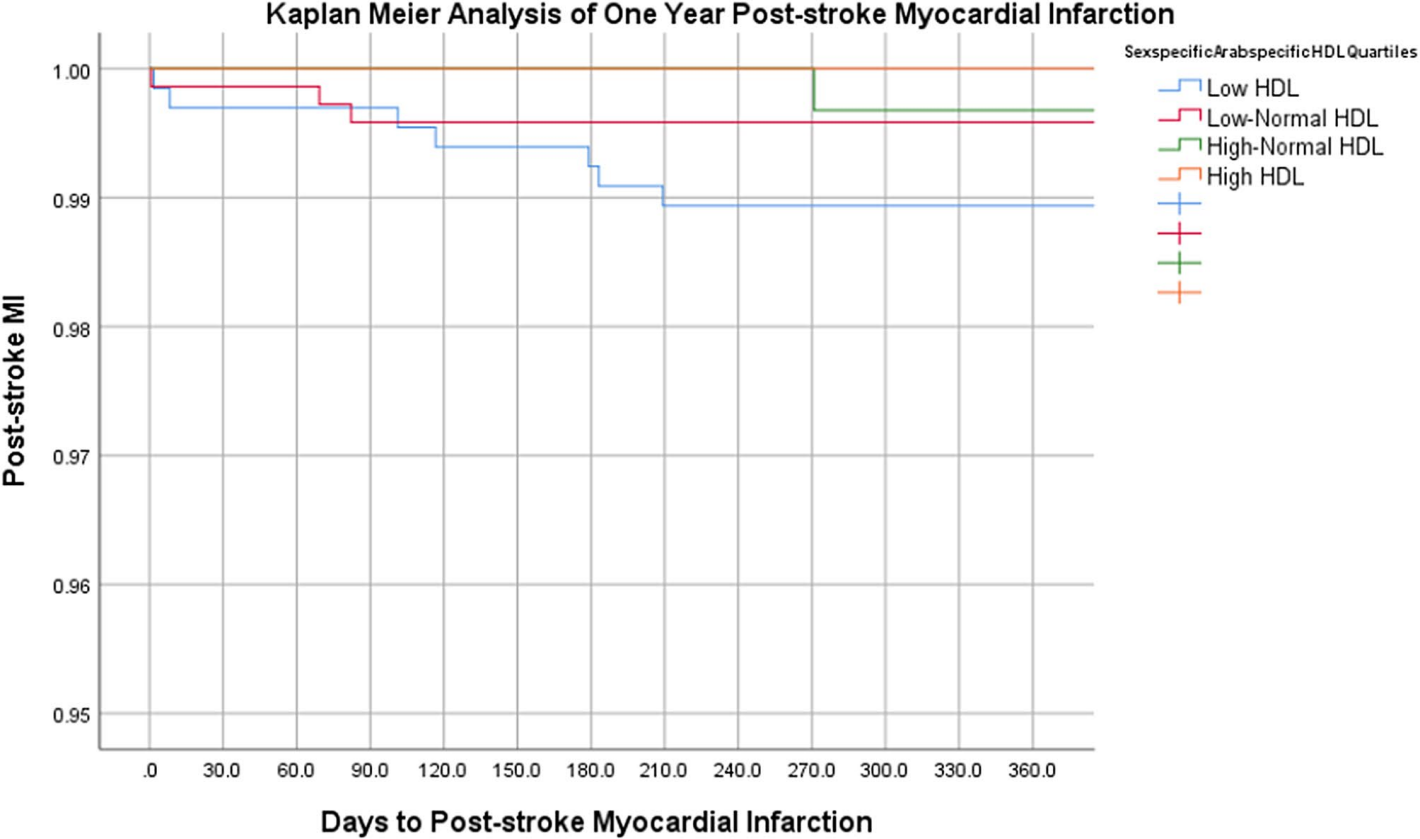
Kaplan meier curve for 1-year post-stroke MI.

On multivariate binary logistic regression analyses, risk adjusted odds ratio for 1-year stroke recurrence was 2.24 times higher (p = 0.034) and for MACE was 1.99 times higher (p = 0.009) in the low-HDL group compared to the high-HDL group. Mortality at 1 year was 2.27-fold in the low-normal HDL group compared to the reference group (p = 0.049). No difference in 90-Day Mortality between the different sex-specific HDL quartiles was observed (Table 3). Table 4 lists a comparative analysis of the outcomes among different studies. Figures 1, 2, 3 represent Kaplan Meier curves for time to recurrent stroke, 1-year post-stroke all cause mortality, and 1-year post-stroke MI.

**Table 3:** Multivariate Binary Logistic Regression Analysis of Outcomes Stratified by Sex-Specific HDL Quartiles.

Sex-specific HDL Cut-offs	Low HDL (n = 658)		Low-Normal HDL (n = 722)		High-Normal HDL (n = 309)		High HDL (n = 400)	
	M: ≤ 0.80 F: ≤ 1.00		M: 0.80–1.00 F: 1.00–1.30		M: 1.00–1.12 F: 1.30–1.60		M: > 1.12 F: > 1.60	
Inpatient outcomes	aOR [95% CI]	*p*	aOR [95% CI]	*p*	aOR [95% CI]	*p*		
In-hospital mortality	0.66 [0.25–1.75]	0.404	0.47 [0.17–1.32]	0.154	0.67 [0.21–2.18]	0.508	Ref	–
In-hospital complications	0.92 [0.56–1.50]	0.725	0.91 [0.56–1.49]	0.721	0.68 [0.37–1.25]	0.210	Ref	–
Long-term outcomes	aOR [95% CI]	*p*	aOR [95% CI]	*p*	aOR [95% CI]	*p*		
1-year stroke recurrence	2.24 [1.07–4.73]	0.034*	1.07 [0.48–2.37]	0.874	1.14 [0.43–2.99]	0.791	Ref	–
90-day mortality	1.82 [0.66–5.02]	0.249	2.11 [0.78–5.71]	0.143	1.89 [0.62–5.81]	0.264	Ref	–
1-year mortality	1.96 [0.85–4.52]	0.114	2.27 [1.03–5.12]	0.049*	2.27 [0.91–5.63]	0.078	Ref	–
1-year post-stroke MACE	1.99 [1.19–3.31]	0.009*	1.44 [0.86–2.42]	0.166	1.55 [0.86–2.80]	0.148	Ref	–
1-year Post-stroke Cardiac Revascularization	1.28 [0.22–7.56]	0.785	1.27 [0.22–7.39]	0.789	2.54 [0.40–16.16]	0.324	Ref	–

*HDL* high-density lipoprotein, *LOS* length of stay, d days, *IQR* interquartile range, *ICU* intensive care unit, *MRS* modified Rankin Scale, *MACE* major adverse cardiac event, *MI* myocardial infarction, *aOR* adjusted odds ratio, *95% CI* 95% confidence interval, *M* males, *F* females.

Multivariate analyses adjusted for patient demographics (age, sex, ethnicity), emergency department vitals (systolic blood pressure), comorbidities (diabetes, hypertension, dyslipidemia, coronary artery disease, atrial fibrillation, prior stroke history, obesity, smoking status), stroke severity, and in-hospital interventions (thrombolysis or thrombectomy).

**Table 4:** Comparison of Post-Stroke Outcomes in Different HDL Quartiles, previously published studies.

Study	Study type, sample size, ethnicity	Follow-up duration	HDL quartiles (mmol/L)	Post-stroke outcomes (MT rate, post-stroke mortality, stroke recurrence, post-stroke MACE)
Our study 2023	Retrospective analysis of prospectively collected data N = 2089 Arab	1 year	L: M ≤ 0.80, F ≤ 1.00	1-year stroke recurrence: aOR 2.24 95% CI 1.07–4.73, p = 0.034* 1-year mortality: aOR 2.27, 95% CI 1.03–5.12, p = 0.049* 1-year post-stroke MACE: aOR 1.99, 95% CI1.19–3.31, p = 0.009* lower HDL-C was associated with higher MT rates 5.2% vs. 2.8%; p = 0.026*
			LN: M: 0.80–1.00, F: 1.00–1.30	
			HN: M: 1.00–1.12 F: 1.30–1.60	
			H: M: > 1.12 (Ref.) F: > 1.60 (Ref.)	
Mei et al. 2014	Prospective N = 1059 Chinese	4 years	H: ≥ 1.55 Moderate:1.04–1.54 L : < 1.04	Stroke recurrence: HDL-C ≤ 1.0 (HR = 1.944, 95% CI:1.033- 3.659, P = 0.039*) HDL-C 1.01—1.19 (HR = 2.027, 95% CI:1.116–3.682, P = 0.020*)
Kuwashiro et al. 2012	Prospective N = 260 Japanese	1 year	H: > 40 mg/dl L: < 40 mg/dl	Stroke recurrence: OR 2.73; 95% CI: 1.01–7.38; p = .048*
C.-W. Liou et al. 2008	Prospective N = 1062 Vietnamese	1 year	L: M: < 40 mg/dl F: < 50 mg/dl	Stroke recurrence: Low HDL (OR, 1.43; 95% CI: 1.08– 1.88, p = 0.011*)
Amarenco et al. 2008	Randomized controlled trial N = 4731 USA	4.9 years	1st tertile: 0.23–1.09 2nd tertile: 1.11–1.37 3rd tertile: 1.39–3.21	Stroke recurrence: By 1 s.d HDL increment (HR = 0.90, 95% CI: 0.82–0.99, p = 0.03*) MACE: By 1 s.d HDL increment (HR = 0.91, 95% CI: 0.83–0.99, p = 0.021*)
Park et al. 2014	Analysis of Randomzied controlled trial data N = 3680 USA, Canada, Scotland	2 years	Data not shown	lowest HDL-C quintile was not associated with Recurrent stroke or MACE after multivariable Cox analyses Data not shown
Vitturi et al. 2021	Prospective N = 588 Brazilian	2 years	L: M: < 40 mg/dl F: < 50 mg/dl	Stroke recurrence: OR 0.76, C.I 0.41–1.39, p = 0.47 MACE: OR 5.96, C.I 1.66–21.39, p = 0.005*
Schwedhelm et al. 2022	Prospective N = 681 German	1 year	H: > 49 mg/dl L: < 49 mg/dl	MACE HR = 0.71, 95% CI 0.30–1.71, p = 0.446
Pedrovic et al. 2016	Prospective N = 110 Croatian	11 Days	Data not shown	Low HDL at discharge was associated with Barthel Index† < 60 OR 0.049, CI 0.003–0.704, p = 0.038*
Tian et al 2013	Cross-sectional N = 1568 Chinese	Hospital discharge	H: > 1.04 mmol/L L: < 1.04 mmol/L	Low HDL was associated with NIHSS > 10 at discharge or death OR 0.482, CI 0.235–0.946
Bravi et al. 2023	Retrospective N = 1639 Italian	5 years	Data not shown	MT patients showed lower levels of Non-HDL-C 117 mg/dl [IQR; 94–145] vs. 127 mg/dl [IQR; 103–154], P < 0.001*

*MT* mechanical thrombectomy, *MACE* major adverse cardiovascular events, *CHD* coronary heart disease, *MI* myocardial infarction, *HDL-C* high density lipoprotein cholesterol, *C.I* confidence interval, *S.D* standard deviation, *H* high, *LN* low-normal, *L* low, *M* male, *F* female.

^†^Barthel Index = Marker of patient disability.

## Discussion

Our study shows that a low HDL was associated with a 2.24-fold risk of 1-year stroke recurrence on multivariate binary logistic regression analysis in the Arab cohort. Several studies have previously shown an inverse relationship between lower HDL and an increased risk of stroke^7–12^. There is however limited data on the relationship between HDL and short and long-term post-stroke outcomes, especially in the Arab population where lipid abnormalities are very common.

Mei et al. conducted a study on 1059 ischemic stroke patients in a Chinese population, and reported two-fold risk of recurrent stroke in the low-HDL group compared to the high HDL group^13^. A Vietnamese study identified low HDL as a significant recurrent ischemic stroke risk factor (OR 1.43; 95% CI 1.08–1.88)^14^. The stroke prevention by aggressive reduction in cholesterol levels (SPARCL) trial similarly showed each 13.7 mg/dl increment in HDL to be associated with a 13% reduction in ischemic stroke risk with reduction in MACE events reported as well^15^. Conversely**,** Park et al. analysed the Vitamin Intervention for Stroke Prevention (VISP) study database comprising of 3680 non-cardioembolic stroke patients followed up for up to 2 years, and was unable to demonstrate a significant association between baseline HDL-C and recurrent ischemic stroke in multi-variable cox analysis. However, the different outcome of this study may be attributed to not being able to adjust for metabolic syndrome due to unavailability of glucose levels^16^.

The risk of MACE was 1.99 times higher in the low-HDL group when compared to the high-HDL group (p = 0.009). Major adverse cardiovascular events (MACE) are common in patients who suffer an acute ischemic stroke^4^, and our study corroborated these findings. This is similar to the report in a Brazilian cohort by Vitturi et al., who demonstrated an inverse association between low HDL-C levels and increased risk of MACE (OR 5.96, 1.66–21.39, p = 0.005), but not increased stroke recurrence^17^. There are other reports where the association was not established^18^.

Mortality at 1 year was however 2.27-fold higher in the low-normal HDL group compared to the reference group (p = 0.049). Perovic et al., observed in a small study of 52 patients that lower HDL at discharge was associated with significantly higher patient disability (Barthel index < 60), and mortality at 90-days^19^. Tian et al. had similar observations in 1568 Chinese ischemic stroke patients, in whom low HDL measured within 24 h of admission was associated with adverse patient outcomes (defined as NIHSS > 10 at discharge or death)^20^.

However**,** low HDL was also associated with almost twice as high thrombectomy rates as compared to high HDL on univariate analysis in our population. Bravi et al. had similar outcomes in an Italian cohort where endovascular thrombectomy (EVT) patients showed lower levels of non-HDL-C, compared to non-EVT patients (117 mg/dl [IQR; 94–145] vs 127 mg/dl [IQR; 103–154], P < 0.001)^21^. Some reasons for our findings could be that low HDL-C has been shown to be associated with a significant increase in carotid plaque volume by ultrasound^22^, and increased infarct size^23^. Another explanation could be the inverse association of HDL levels with development of intracranial atherosclerotic stenosis (ICAS), severity of ICAS, and multi-vessel involvement of ICAS^24^**.**

Our findings can be attributed to the several mechanisms by which HDL protects against atherosclerosis. It transports cholesterol from the artery wall to the liver for excretion by the mechanism of reverse cholesterol transport. It blocks inflammation by acting as an anti-oxidant and it reduces thrombotic risk by inhibition of platelet activation and aggregation. HDL has been able to reduce neuronal damage after onset of ischemic stroke in both excitotoxic and MCA occlusion models of stroke^22^. Interestingly, Experimental IV infusion of HDL has shown neuroprotective effects by reducing lesion size^25^. Moreover, apolipoprotein A-1 (Apo A-1), which is a component of HDL, has been associated with a decrease of brain lesion size by 64% in a middle cerebral artery occlusion model^22^. HDL has also been shown to enhance insulin sensitivity and promote insulin secretion by pancreatic beta islet cells^24^.

A number of clinical trials that involved HDL-C raising strategies, including AIM-HIGH trial (niacin), ACCORD trial (fenofibrate), and ILLUMINATE trial (torcetrapib), however, failed to demonstrate any clinical benefits with the treatments^24^. A reasonable explanation why the treatment-induced increase in HDL does not result in positive results may be dysfunctional HDL, similar to the HDL patients with DM, CKD, or metabolic syndrome have. It has been proposed that this may lead to endothelial injury and promote atherosclerotic processes. Edzard Schwedhelm proposed calculating the 'biologically effective’ HDL cholesterol level as a way to differentiate between regular and dysfunctional HDL^18^. Multiple observational studies have also found that HDL particle number and size may be better predictors of cardiovascular disease than HDL- C alone^11^.

## Conclusion

Our study shows that lower sex-specific HDL levels were associated with worse 1-year post-stroke neurologic and also cardiovascular outcomes. This is the first reported corroboration of previously known associations between HDL levels and long-term stroke outcomes in the Arab population. Our findings highlight the importance of cardiovascular risk stratification after initial stroke occurrence. These interventions may play an even more important role in the Arab population where dyslipidemia is highly prevalent. The 90-Day mRS showed no difference between the different HDL quartiles but at the 1-year visit, a difference in mortality was observed. Supplementing the 90-Day clinic visit with 1-year and 5-year follow-up visits will lead to more accurate representation of post-stroke outcomes.

## Limitations

Only baseline lipid variables were included. Subtypes of HDL were not studied. Since our patient population was mainly Arabs, our study cannot be generalized to other ethnic populations. As two-third of the study population was male, we will not be able to extrapolate these findings to Arab females. No data on alcohol consumption was available as it is in a Qatari population, where alcohol consumption is prohibited.

## Data Availability

Original data is available and can be accessed by direct request from Dr Naveed Akhtar at naveedakhtars@hotmail.com.
